# Effects of mental fatigue on lower-limb explosive power and anaerobic capacity: a systematic review and meta-analysis

**DOI:** 10.3389/fphys.2026.1916804

**Published:** 2026-09-23

**Authors:** Ning Wang, Zhengxiang Chen, Kewei Zhao

**Affiliations:** China Institute of Sport Science, Beijing, China

**Keywords:** anaerobic capacity, lower-limb explosive power, mental fatigue, meta-analysis, systematic review

## Abstract

**Introduction:**

Mental fatigue may impair high-intensity exercise performance, but its effects on lower-limb explosive power and anaerobic capacity remain inconsistent. This study aimed to systematically evaluate and quantitatively synthesize the effects of mental fatigue on these two performance domains.

**Methods:**

This study was conducted according to the PRISMA 2020 guidelines. PubMed, Web of Science, Scopus, and CNKI were searched for randomized controlled trials and randomized crossover trials. Standardized mean differences were calculated using Hedges’ g, and pooled effects were estimated using a random-effects model. Risk-of-bias assessment, sensitivity analyses, and subgroup analyses were also conducted.

**Results:**

A total of 17 studies involving 302 participants were included. Mental fatigue significantly reduced lower-limb explosive power, although the effect size was small and heterogeneity was low (SMD = −0.16, 95% CI [−0.28, −0.03], p = 0.014). Mental fatigue also significantly impaired anaerobic capacity (SMD = −0.29, 95% CI [−0.52, −0.06], p = 0.013). No statistically significant between-subgroup differences were observed for athletic level, mental-fatigue induction duration, or sport specificity of the performance test.

**Conclusion:**

Mental fatigue appears to impair both lower-limb explosive power and anaerobic capacity, although the anaerobic-capacity estimate should be interpreted cautiously because of imprecision and lower certainty of evidence. Cognitive load management should therefore be considered in training design and pre-competition preparation.

**Systematic Review Registration:**

https://www.crd.york.ac.uk/PROSPERO/view/CRD42025116370, identifier CRD42025116370.

## 1 Introduction

Mental fatigue is a complex psychobiological state induced by prolonged tasks with high cognitive demands. It is characterized by increased subjective feelings of exhaustion, reduced motivation, and objective declines in cognitive function (Boksem and Tops, 2008; Steward et al., 2025). Recent theoretical evidence further suggests that cognitive fatigue is not merely a transient feeling of tiredness but a multidimensional state involving altered motivation, effort allocation, cognitive control, and decision-making efficiency (Pessiglione et al., 2025). In modern high-intensity competitive sports, athletes must continuously engage in tactical anticipation and self-regulation; the accumulation of cognitive load has made mental fatigue an important factor influencing athletic performance (Habay et al., 2021; Lu et al., 2026).

Over the past decade, previous evidence has generally shown that mental fatigue impairs aerobic endurance performance (Van Cutsem et al., 2017; Brown et al., 2020). Several studies have reported that cognitive exertion is followed by a shorter time to exhaustion and an overall decline in endurance performance. According to Marcora et al., mental fatigue does not arise from peripheral physiological failure but may amplify perceived exertion (RPE), prompting the central nervous system to reduce intensity or terminate exercise prematurely (Marcora, 2008; Marcora et al., 2009). However, these theoretical explanations may not fully apply to extremely short-duration, high-intensity anaerobic and explosive exercise tasks. Lower-limb explosive power and anaerobic capacity are important determinants of performance in many sports (Nikolaidis et al., 2016; Banda et al., 2019; Tan et al., 2026; Xu et al., 2026). Unlike endurance exercise, extremely high-intensity exercise is primarily supported by the phosphagen and glycolytic energy systems and relies heavily on the rapid recruitment of motor units by the central nervous system (Gandevia, 2001; Baker et al., 2010; Cormie et al., 2011). Whether cognitive impairment caused by mental fatigue can substantially interfere with this rapid neuromuscular control remains inconclusive.

Regarding the effects of mental fatigue on lower-limb explosive power and anaerobic capacity, findings in the existing literature remain inconsistent. Some studies suggest that mental fatigue reduces excitability in the prefrontal cortex and motor cortex, thereby weakening neural drive to muscles and ultimately reducing power output or slowing sprint speed (Mehta and Parasuraman, 2014; Smith et al., 2015; Staiano et al., 2024). However, other studies have reported contrasting findings, suggesting that maximal force exerted over extremely brief durations, such as a single vertical jump or a very short-distance sprint, is largely unaffected by mental fatigue (Martin et al., 2018; Alix-Fages et al., 2023; Fortes et al., 2024). Several factors may explain these inconsistent findings. First, individual empirical studies generally have small sample sizes, which limits statistical power (Turner et al., 2013; Halperin et al., 2018). Second, substantial heterogeneity exists in experimental designs. The cognitive tasks used to induce fatigue, such as Stroop and AX-CPT tasks, and their durations, ranging from 15 to 90 min (Lorist et al., 2005; Boksem and Tops, 2008; Hassan et al., 2023), vary across studies. Recent methodological work has also emphasized that many traditional mental-fatigue paradigms rely heavily on subjective fatigue ratings and repetitive laboratory tasks, whereas more ecologically valid protocols and objective indicators of fatigue are still needed (Hassan et al., 2023). Participants also differ in training background, and studies use different performance tests, such as single-leg vertical jumps and Wingate or repeated-sprint tests (Filipas et al., 2019; Scharfen and Memmert, 2019). Exercise-task characteristics, participant profiles, and cognitive-intervention details may therefore act as potential moderators (Russell et al., 2019; Habay et al., 2021).

Although systematic reviews have explored the effects of mental fatigue on athletic performance, the existing evidence remains limited. First, previous reviews have often synthesized different exercise tasks, including endurance, strength, technical performance, and intermittent exercise performance, and few have separately analyzed high-intensity, short-duration outcomes. Second, current findings on the effects of mental fatigue on lower-limb explosive power and anaerobic capacity remain inconsistent. Individual studies generally have small samples, varied test metrics, and participants with different training backgrounds, which limits the interpretation and generalizability of their results. Lower-limb explosive power and anaerobic capacity are crucial physical capacities for initiating movement, jumping, sprinting, changing direction, and sustaining repeated high-intensity output across many sports. Therefore, a systematic review and meta-analysis is needed to quantitatively synthesize the existing evidence.

Therefore, this systematic review and meta-analysis aimed to identify, screen, and quantitatively synthesize relevant studies assessing the effects of mental fatigue on lower-limb explosive power and anaerobic capacity in healthy individuals and athletes. Based on theories of neuromuscular control, central fatigue, and subjective effort regulation, we hypothesized that mental fatigue would have a limited effect on lower-limb explosive power, which is extremely short in duration, relies primarily on phosphagen energy supply, and involves rapid neuromuscular recruitment. We expected a more pronounced adverse effect on anaerobic capacity, which involves relatively longer durations, sustained high-intensity output, and greater metabolic stress. Subgroup analyses were conducted to explore whether participants’ training level, cognitive-task type, induction duration, and exercise-test format may influence the effects of mental fatigue. The findings may provide more targeted evidence for cognitive-load management, pre-competition preparation, and training planning in high-intensity sports.

## 2 Methods

### 2.1 Protocol and registration

This systematic review and meta-analysis was conducted and reported in accordance with the Preferred Reporting Items for Systematic Reviews and Meta-Analyses 2020 statement (Page et al., 2021). The protocol was preregistered in PROSPERO before the literature search was conducted, with the registration number CRD42025116370.

### 2.2 Search strategy

Two independent researchers conducted a comprehensive computerized search of four electronic databases: PubMed, Web of Science, Scopus, and China National Knowledge Infrastructure (CNKI). The search covered all records from database inception to August 2026. The search strategy combined controlled vocabulary and free-text terms using Boolean operators (AND and OR).

Search terms for the English-language databases focused on two concept clusters: (1) mental fatigue (“mental fatigue,” “cognitive fatigue,” and “mental exertion”) and (2) lower-limb explosive power and anaerobic capacity (“explosive power,” “vertical jump,” “CMJ,” “anaerobic,” “Wingate,” and “sprint,” among others). For CNKI, corresponding Chinese keywords were used to search the title, abstract, and keyword fields. The detailed search strategy is available in **Supplementary Table S1**.

To minimize the risk of omitting relevant literature, citation tracking of the included studies and related systematic reviews was conducted to identify potentially eligible studies. Reference lists of included studies and relevant reviews were manually screened, and no restrictions were imposed on publication language. Grey literature and unpublished studies were not systematically searched, which may have introduced publication bias.

### 2.3 Inclusion and exclusion criteria

The PICOS framework was used to assess study eligibility. Titles and abstracts were screened, followed by full-text evaluation. Eligibility was determined according to the PICOS framework (population, intervention, control condition, outcomes, and study design), as summarized in **Table 1**.

**Table 1:** Eligibility criteria based on the PICOS framework.

Dimension	Inclusion criteria	Exclusion criteria
Participants	Healthy individuals aged ≥ 16 years (including professional athletes, recreational athletes, and the general adult population); no sex restrictions.	Age < 16 years; individuals with neurological or psychiatric disorders, or those with a recent history of severe lower-limb sports injuries.
Intervention	Induction of mental fatigue using well-established, prolonged cognitive tasks (e.g., Stroop, AX-CPT, TMT, or simulated game analysis).	Combined fatigue interventions (i.e., tasks that simultaneously induce mental and physical fatigue, making it impossible to isolate the specific mental effects).
Control condition	A matched control condition with low cognitive load.	Absence of a control-condition design or use of a control condition with high cognitive load.
Outcome measures	Lower-limb explosive power: jump height (CMJ/SJ/vertical jump), peak power Anaerobic capacity: all-out cycling, sprint/repeated-sprint, and repeated-jump/power-endurance performance	Studies that reported only subjective fatigue scales (e.g., RPE, VAS, or NASA-TLX) without objective performance measures.
Study design	Randomized controlled trials (RCTs) or randomized crossover trials.	Reviews, conference abstracts, case reports, non-randomized studies, and animal experiments.

AX-CPT, AX Continuous Performance Task; CMJ, countermovement jump; NASA-TLX, NASA Task Load Index; RPE, rating of perceived exertion; SJ, squat jump; TMT, Trail Making Test; VAS, visual analogue scale.

For the purposes of the present synthesis, anaerobic-capacity outcomes were defined as measures of maximal or repeated high-intensity performance. Measures primarily reflecting agility, change-of-direction ability, balance, technical skill, or endurance were classified outside the anaerobic-capacity domain. Study-level inclusion did not imply inclusion of all reported outcomes; each outcome was assessed separately for eligibility within the relevant synthesis.

### 2.4 Literature screening and data extraction

#### 2.4.1 Literature screening

All records retrieved from electronic databases were imported into Zotero reference management software for deduplication. Screening was conducted independently by two researchers (N.W. and Z.X.C.). First, clearly ineligible studies were excluded based on titles and abstracts. The full texts of the remaining reports were then retrieved and assessed to determine final eligibility. Inter-rater agreement was assessed using Cohen’s kappa coefficient (Cohen, 1988). Disagreements were resolved through discussion; if consensus could not be reached, a third senior researcher (K.W.Z.) made the final decision.

#### 2.4.2 Data extraction

Two researchers independently extracted data using a standardized Microsoft Excel spreadsheet and cross-checked the results. The extracted information included basic study characteristics (first author, publication year, and source); participant characteristics (sample size, sex, and training level or population type); mental fatigue induction protocols (cognitive-task type and induction duration); performance-test metrics (lower-limb explosive power and anaerobic capacity tests); and outcome data (means, standard deviations, and sample sizes under mental fatigue and control conditions).

For studies that presented data only in graphical form, WebPlotDigitizer was used to extract numerical values (Drevon et al., 2017). If standard errors (SEs) or 95% confidence intervals (CIs) were reported, they were converted to standard deviations (SDs) using standard statistical formulas. Given the widespread use of randomized crossover designs in this field, paired effect estimates comparing mental fatigue and control conditions were extracted whenever possible. For studies that did not report within-participant correlation coefficients between conditions, a conservative correlation coefficient of 0.5 was assumed in the primary data synthesis, and the robustness of this assumption was examined through sensitivity analyses (Elbourne et al., 2002; Higgins et al., 2019).

### 2.5 Risk of bias assessment

The risk of bias of the included studies was assessed independently by two researchers using the Cochrane Risk of Bias tool (Higgins et al., 2011). Because the included studies were primarily randomized controlled trials or randomized crossover trials, seven domains were assessed: (1) random sequence generation; (2) allocation concealment; (3) blinding of participants and personnel; (4) blinding of outcome assessment; (5) incomplete outcome data; (6) selective reporting; and (7) other potential sources of bias. Each domain was rated as low, unclear, or high risk. Discrepancies were resolved through discussion, with a third senior researcher making the final determination when necessary. Summary tables and traffic-light plots were visualized using the robvis tool (McGuinness and Higgins, 2021).

### 2.6 Sensitivity analysis

Given that most included studies were crossover trials, sensitivity analyses were conducted for studies in which a conservative correlation coefficient (r = 0.5) was assumed because the original report did not provide one. Variances and effect sizes were recalculated using alternative correlation coefficients of r = 0.3 and r = 0.7 to assess whether this assumption materially altered the conclusions. Funnel plots were constructed for visual inspection of potential publication bias or small-study effects. Funnel plots displayed effect size on the x-axis and standard error on the y-axis. Egger’s regression test was performed only when at least 10 independent effect sizes were available for a primary outcome, with p < 0.05 indicating potential asymmetry (Sterne et al., 2019). A leave-one-out sensitivity analysis was also conducted by sequentially excluding each effect size and refitting the REML random-effects model. Changes in the pooled Hedges’ g, 95% CI, statistical significance, I^2^, and τ^2^ were examined to assess the influence of individual studies.

### 2.7 Certainty of evidence assessment

The certainty of evidence for each primary outcome was assessed using the Grading of Recommendations Assessment, Development and Evaluation (GRADE) approach (Guyatt et al., 2008). Two reviewers independently rated the certainty of evidence for lower-limb explosive power and anaerobic capacity as high, moderate, low, or very low. Randomized controlled trials and randomized crossover trials were initially considered high-certainty evidence. The certainty of evidence was downgraded based on five domains: risk of bias, inconsistency, indirectness, imprecision, and publication bias. Disagreements were resolved through discussion or consultation with a third reviewer. A summary-of-findings table was constructed to present the pooled effect estimates, number of included studies and participants, heterogeneity, and certainty ratings for each outcome.

### 2.8 Statistical analysis

All statistical analyses were performed using R software with the meta and metafor packages (Viechtbauer, 2010; Balduzzi et al., 2019). Because the included studies used different testing instruments and measurement units for lower-limb explosive power (e.g., squat-jump height and power output) and anaerobic capacity (e.g., peak power), standardized mean differences (SMDs) and their 95% confidence intervals (CIs) were used as pooled effect sizes. Hedges’ g was applied to correct for small-sample bias. Effect sizes were interpreted as small, moderate, and large at thresholds of 0.2, 0.5, and 0.8, respectively (Cohen, 2013). For outcomes in which lower values indicated better performance, such as sprint time, repeated-sprint time, effect sizes were reverse-coded so that negative Hedges’ g values consistently indicated impaired performance after mental fatigue. Statistical heterogeneity was assessed using Cochran’s Q test (p < 0.10) and the I^2^ statistic. Given the expected clinical and methodological differences among studies, a random-effects model based on restricted maximum-likelihood estimation (REML) was applied. Prespecified subgroup analyses explored participant training level (non-athletes, collegiate athletes, youth athletes, and elite athletes), cognitive-task type (Stroop task, social media use, and other tasks), mental fatigue induction duration (≤45 min vs. >45 min), jump-test type (CMJ, SJ, and other jump tests), and sport specificity of anaerobic capacity testing (sport-specific vs. non-sport-specific tests).

## 3 Results

### 3.1 Study selection

Database searches identified 2,718 records. Of these, 31 reports were sought for retrieval, seven of which were unavailable. Twenty-four reports were assessed for eligibility, and seven reports were excluded after full-text assessment. A total of 17 studies were included in the systematic review. Among these, 16 contributed to the lower-limb explosive-power analysis, 9 contributed to the anaerobic-capacity analysis, and 8 contributed to both outcome domains. The study selection process is summarized in **Figure 1**.

**Figure 1:**
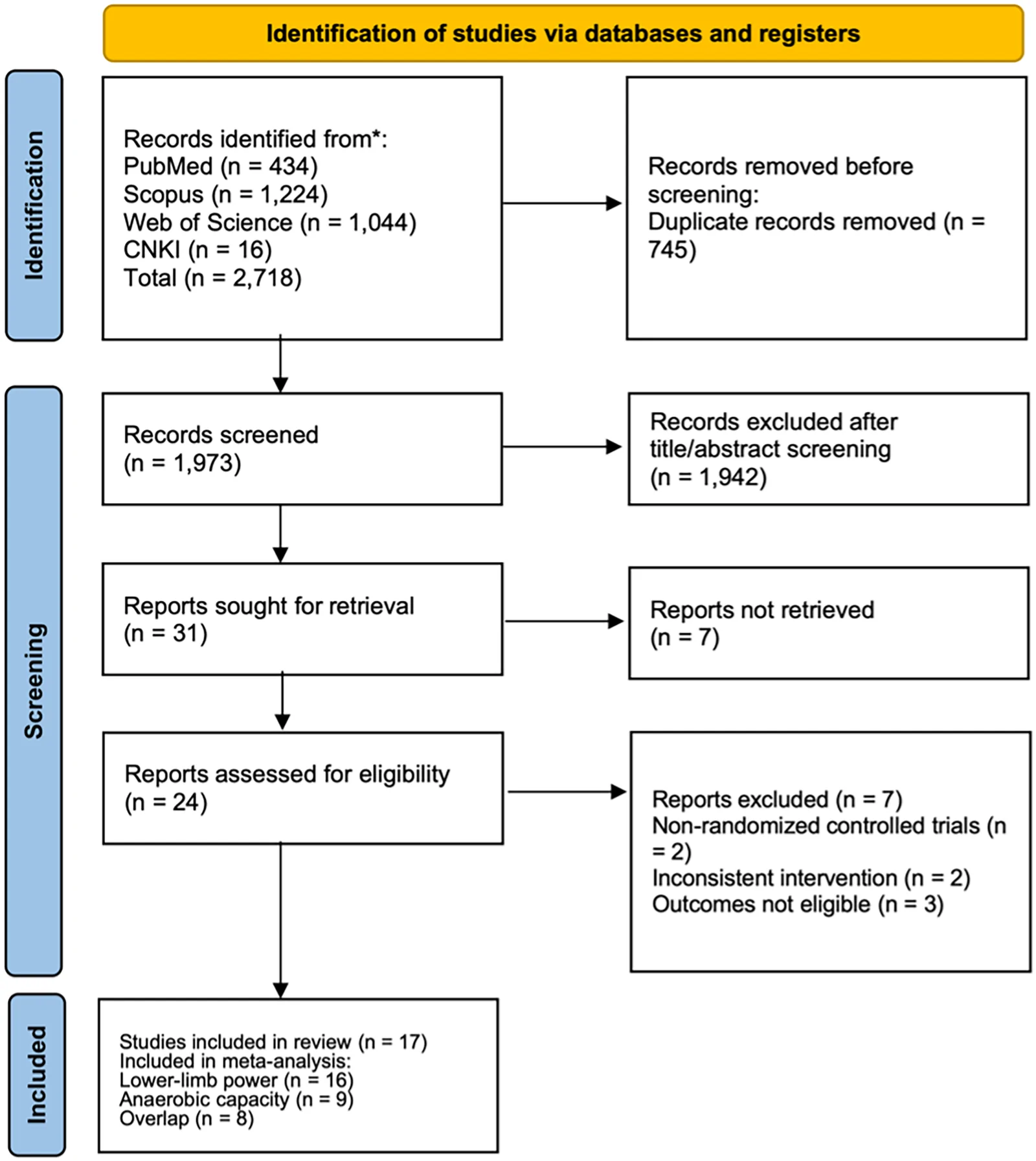
PRISMA flow diagram.

### 3.2 Study characteristics

The basic characteristics of the included studies are shown in **Table 2**. A total of 17 studies involving 302 participants were included. Participants were primarily healthy adults and young athletes; most studies recruited male participants, while some included mixed-sex samples. Participants included collegiate athletes, elite athletes, youth athletes, and non-athletes. Most studies used the Stroop task to induce mental fatigue, while several used other cognitive-load tasks, such as the AX-CPT or social media use. Induction duration ranged from 20 to 90 min; 30 min was the most common duration, followed by 60 and 90 min.

**Table 2:** Characteristics of the included studies.

Study	N	Sex	Athletic level/population	Cognitive task	Duration	Power test	Anaerobic test
Wang et al., 2025	15	Men	Professional/collegiate American football players	Stroop task	45 min	SJ, CMJ, DJ	–
Alix-Fages et al., 2023	25	Men	Resistance-trained men	Social media use; Stroop task	30 min	CMJ	–
Evans et al., 2025	10	Men	Collegiate indoor cricket players	Smartphone Stroop task; cricket video streaming	30 min	–	English Cricket Board Run-2 sprint test
Fortes et al., 2021	24	Men	Young volleyball athletes	Social media use	30 min	CMJ	–
Stafylidis et al., 2025	22	12 men/10 women	Physically active young adults	SOMA-NPT mobile-app Stroop task	20 min	Squat jump	–
Martin et al., 2015	12	7 men/5 women	High-intensity trained adults	AX-CPT	90 min	CMJ	3-minute all-out cycling test
de Queiros et al., 2021	9	Men	Resistance-trained collegiate men	Stroop task	30 min	CMJ	Half-squat sets to concentric failure
Verschueren et al., 2020	14	10 men/4 women	Healthy adults	Incongruent Stroop task	90 min	Single-leg hop; CMJ	–
Staiano et al., 2024	18	Men	Amateur team-sport athletes	Incongruent Stroop task	30 min	CMJ; repeated CMJ/RJA	RSA test: 12 × 20-m sprints
Hemmat et al., 2025	20	Men	Recreationally trained ball players	Stroop task	30 min	Vertical jump	–
Weerakkody et al., 2021	25	Men	Amateur Australian football players	Stroop task	30 min (approx. 35 min incl. intervals)	Standing vertical jump; running vertical jump	–
Gonzalez et al., 2024 Exp 1	14	Mixed; sex distribution NR	Collegiate volleyball athletes	Computerized Stroop task	30 min	Maximal-power squat jump	15 repeated CMJs
Gonzalez et al., 2024 Exp 2	27	Mixed; sex distribution NR	Recreationally active college students	Computerized Stroop task	30 min	Maximal-power squat jump	15 repeated CMJs
Fortes et al., 2024	16	Men	Collegiate sprinters	Social media use; Stroop task	60 min	MPV in half-squat; CMJ	100-m and 200-m sprint
Kosack et al., 2020	19	Men	Elite Danish badminton players	Incongruent Stroop task	60 min	CMJ	Badminton-specific test (BST)
Yang et al., 2022	18	Men	Amateur soccer players	Stroop task	45 min	CMJ	–
Freitas-Junior et al., 2025	14	Men	Young national-level volleyball players	Social media use	30 min	Repeated vertical jump ability (IJT60/RVJA)	Intermittent vertical jump test (4 × 15 s)

AFL, Australian Football League; AX-CPT, AX Continuous Performance Task; BST, badminton-specific test; CMJ, countermovement jump; DJ, drop jump; IJT60, 60-s intermittent vertical jump test; MPV, mean propulsive velocity; NR, not reported; RJA, repeated-jump ability; RSA, repeated-sprint ability; RVJA, repeated vertical jump ability; SJ, squat jump; SOMA-NPT, Soma Neuro Performance Training.

Lower-limb explosive power measures primarily included the countermovement jump (CMJ), squat jump (SJ), vertical jump height (VJH), and repeated vertical jump ability (RVJA). Anaerobic capacity measures primarily included short-distance sprints, repeated-sprint ability (RSA), and repeated half-squat jumps. Overall, 16 studies included lower-limb explosive power outcomes, 9 included anaerobic capacity outcomes, and 8 reported outcomes in both domains.

### 3.3 Risk-of-bias assessment

The Cochrane Risk of Bias tool was used to assess the included studies, and the robvis tool was used to generate risk-of-bias summary plots and traffic-light plots. Overall, incomplete outcome data were generally well controlled. However, substantial uncertainty remained regarding allocation concealment, blinding of participants and personnel, selective reporting, and other potential sources of bias. Because strict blinding is difficult to achieve in mental fatigue interventions, most studies were rated as having high or unclear risk of performance bias.

#### 3.3.1 Lower-limb explosive power (n = 16)

For lower-limb explosive power outcomes, 16 studies were assessed for risk of bias (**Figures 2A, B**). Random sequence generation was rated as low risk in nine studies, unclear risk in six studies, and high risk in one study. Allocation concealment was rated as unclear risk in 15 studies and high risk in one study because relevant methodological details were insufficiently reported. For blinding of participants and personnel, 12 studies were rated as high risk and four as unclear risk. Blinding of outcome assessment was rated as low risk in eight studies, unclear risk in seven studies, and high risk in one study. Incomplete outcome data were generally well controlled, with 11 studies rated as low risk and five as unclear risk. Selective reporting was rated as low risk in one study and unclear risk in 15 studies. Other sources of bias were rated as unclear risk in all 16 studies because preregistered protocols, prespecified outcomes, washout periods, order effects, carryover effects, learning effects, and expectancy effects were not consistently reported.

**Figure 2:**
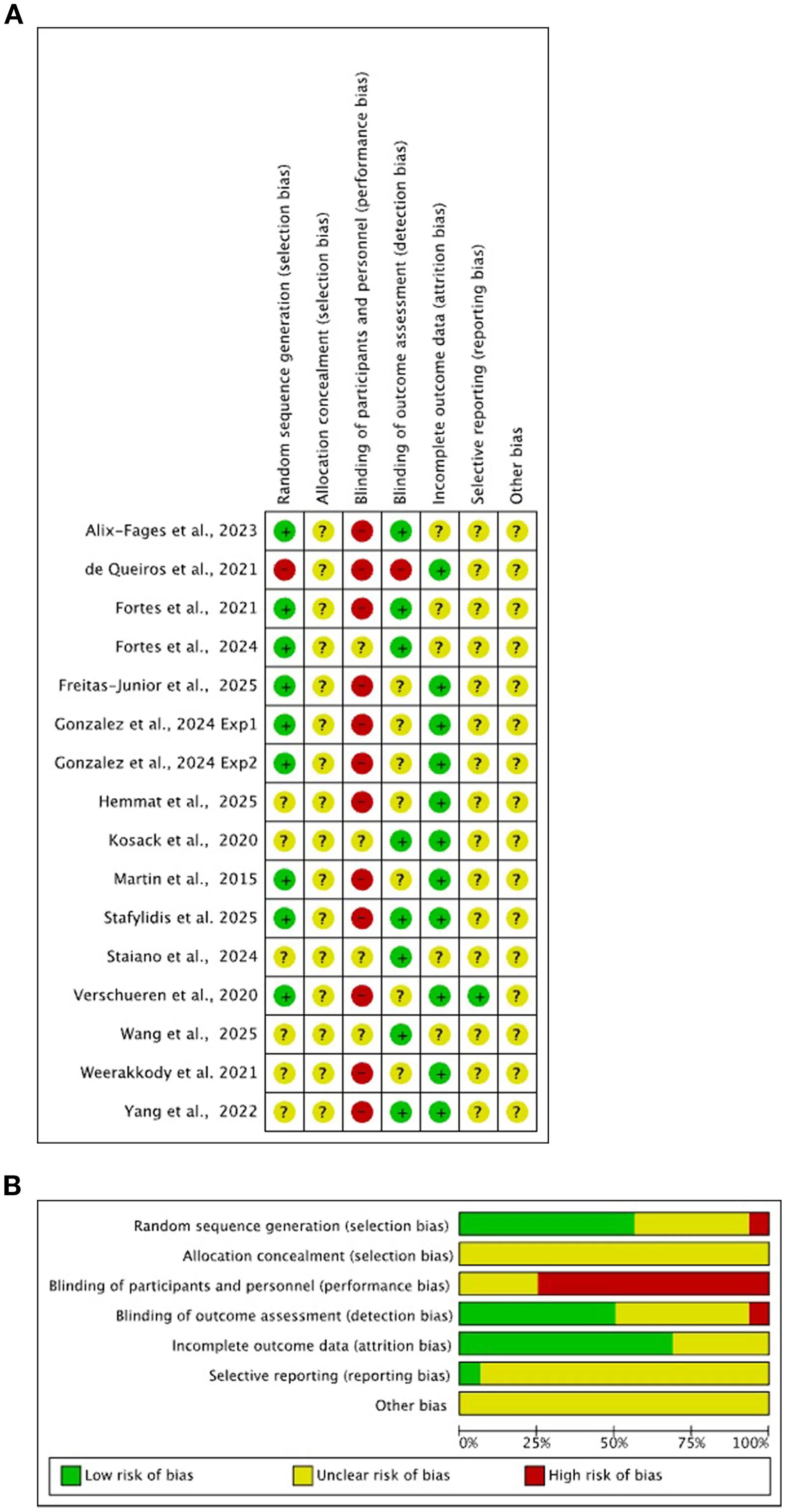
**(A)** Risk-of-bias summary for studies assessing lower-limb explosive power. **(B)** Risk-of-bias graph for studies assessing lower-limb explosive power.

#### 3.3.2 Anaerobic capacity (n = 9)

For anaerobic-capacity outcomes, nine independent experiment-level results were assessed for risk of bias (**Figures 3A, B**). Random sequence generation was rated as low risk in five results, unclear risk in three results, and high risk in one result. Allocation concealment was rated as unclear risk in all nine results because relevant methodological details were insufficiently reported. For blinding of participants and personnel, seven results were rated as high risk and two as unclear risk, reflecting the practical difficulty of blinding participants to mental-fatigue interventions. Blinding of outcome assessment was rated as low risk in three results, unclear risk in five results, and high risk in one result. Incomplete outcome data were generally well controlled, with seven results rated as low risk and two as unclear risk. Selective reporting was rated as unclear risk in all nine results. Other potential sources of bias were also rated as unclear risk in all nine results because preregistered protocols, prespecified outcomes, washout periods, order effects, carryover effects, learning effects, and expectancy effects were not consistently reported. Overall, the principal methodological concerns were related to allocation concealment, blinding of participants and personnel, selective reporting, and insufficient reporting of crossover-specific methodological details.

**Figure 3:**
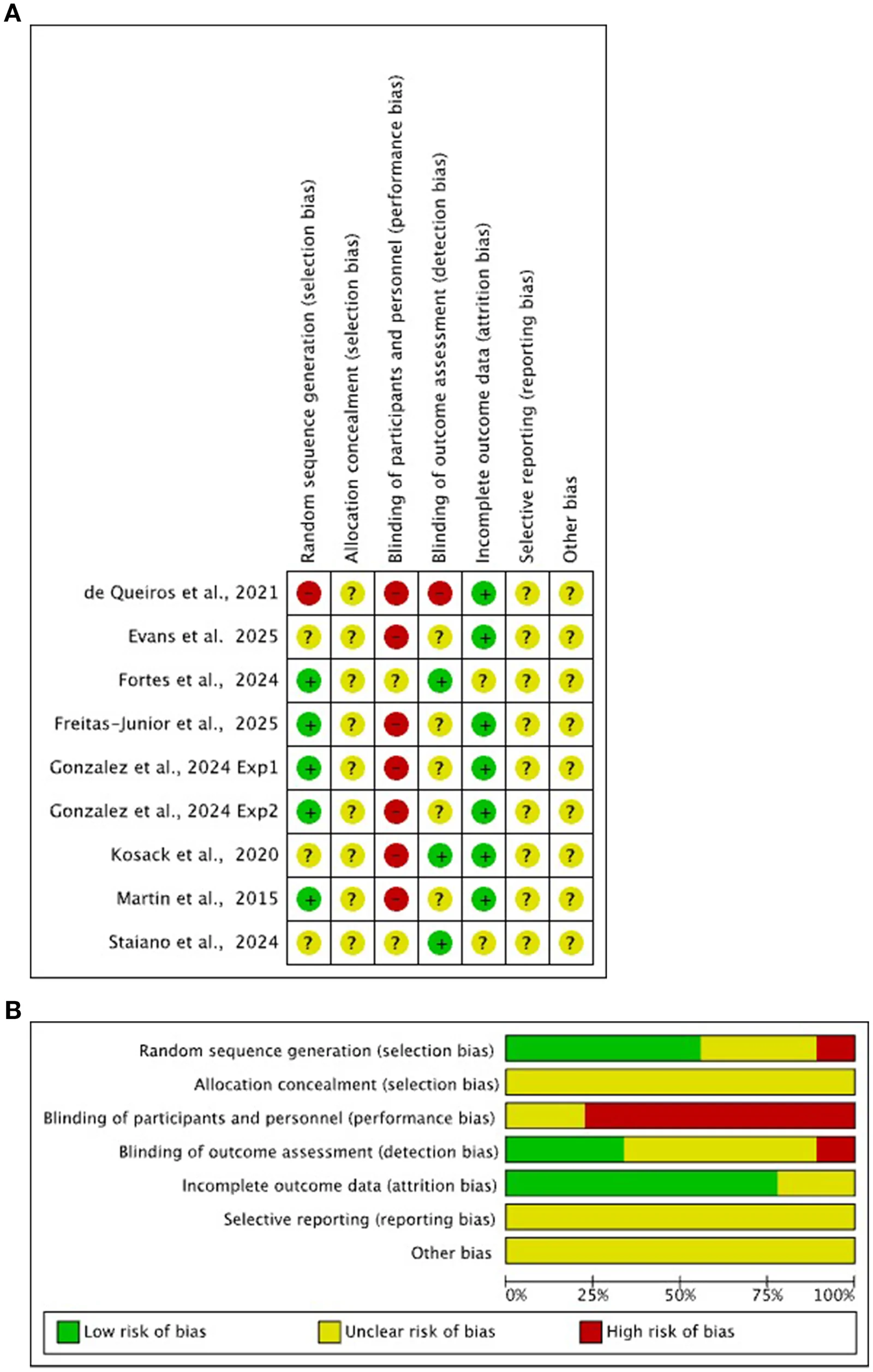
**(A)** Risk-of-bias summary for studies assessing anaerobic capacity. **(B)** Risk-of-bias graph for studies assessing anaerobic capacity.

### 3.4 Sensitivity analysis

To test the robustness of the main effects, sensitivity analyses examined alternative correlation coefficient assumptions and potential funnel plot asymmetry.

#### 3.4.1 Alternative correlation coefficient assumptions

To examine the impact of different assumed within-participant correlations on the pooled estimates, effect sizes and variances were recalculated using r = 0.3, 0.5, and 0.7. In the primary analysis (r = 0.5), the pooled effect size for lower-limb explosive power was Hedges’ g = −0.16, 95% CI [−0.28, −0.03], p = 0.014, with I^2^ = 9.3%. For anaerobic capacity, the pooled effect size was Hedges’ g = −0.29, 95% CI [−0.52, −0.06], p = 0.013, with I^2^ = 43.0%.

For lower-limb explosive power, the pooled effect size was Hedges’ g = −0.16, 95% CI [−0.30, −0.02], p = 0.030, with I^2^ = 0.0% when r = 0.3, and Hedges’ g = −0.16, 95% CI [−0.28, −0.03], p = 0.016, with I^2^ = 40.2% when r = 0.7. For anaerobic capacity, the pooled effect size was Hedges’ g = −0.29, 95% CI [−0.52, −0.06], p = 0.013, with I^2^ = 25.2% when r = 0.3, and Hedges’ g = −0.28, 95% CI [−0.51, −0.06], p = 0.013, with I^2^ = 60.7% when r = 0.7. Across the alternative correlation assumptions, the pooled effect estimates remained highly consistent in direction and magnitude for both outcomes, indicating that the main effect estimates were robust to the assumed within-participant correlation. In contrast, the heterogeneity estimates were more sensitive to the correlation assumption. For lower-limb explosive power, I^2^ ranged from 0.0% to 40.2%, whereas for anaerobic capacity, I^2^ ranged from 25.2% to 60.7%. Thus, although the primary conclusions regarding the pooled effects remained unchanged, the precise magnitude of between-study heterogeneity should be interpreted cautiously.

#### 3.4.2 Funnel plot and Egger’s test

For lower-limb explosive power, Egger’s regression test did not indicate statistically significant funnel plot asymmetry (t = −0.828, p = 0.421). For anaerobic capacity, Egger’s regression test was not performed because fewer than 10 independent effect sizes were available (k = 9). Funnel plots (**Figures 4A, B**) were visually inspected; however, given the limited number of studies, potential publication bias or small-study effects cannot be ruled out.

**Figure 4:**
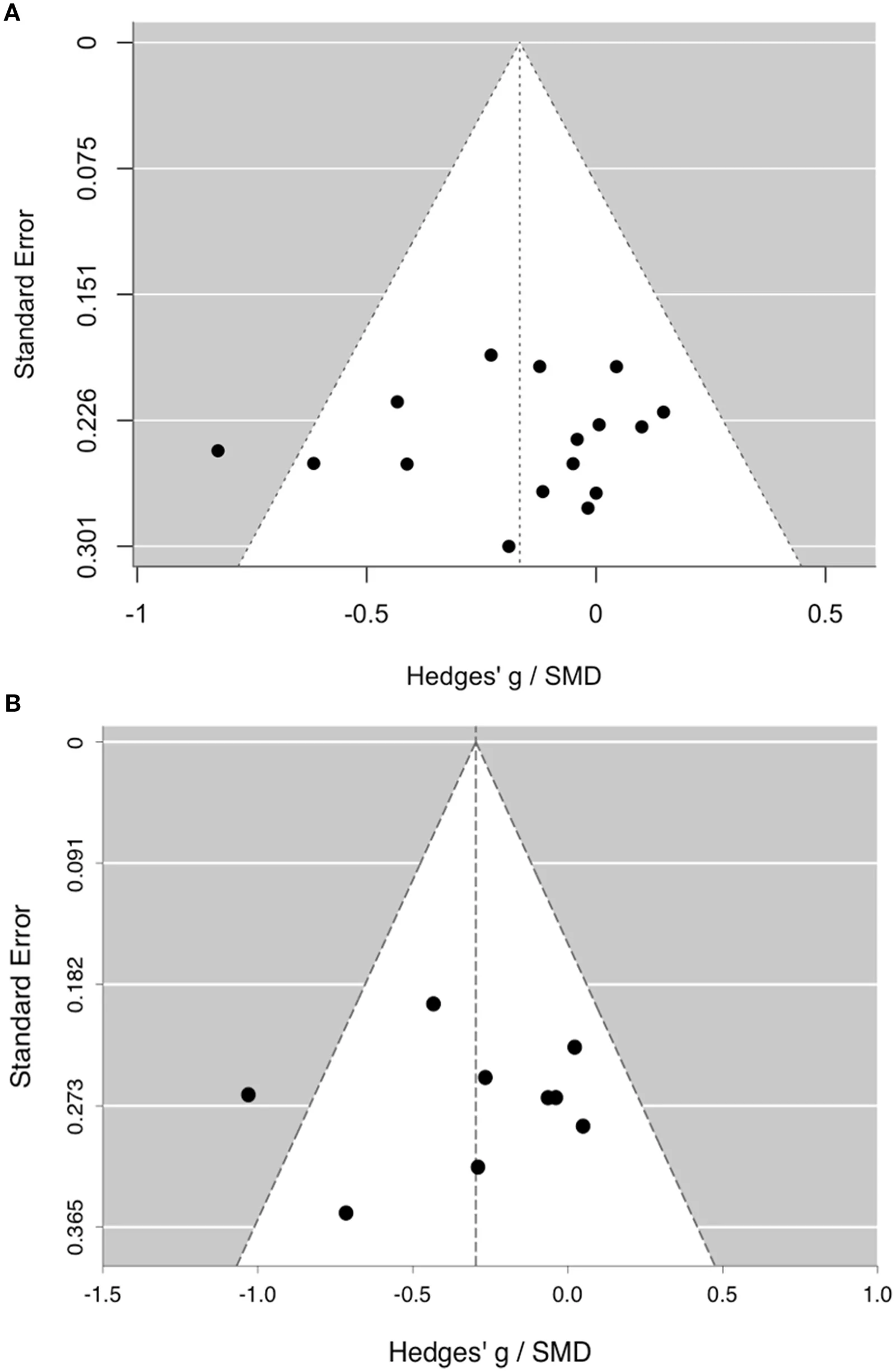
**(A)** Funnel plot for lower-limb explosive power. **(B)** Funnel plot for anaerobic capacity.

#### 3.4.3 Leave-one-out sensitivity analysis

An additional leave-one-out sensitivity analysis was conducted for the anaerobic-capacity synthesis. Across the sequential exclusion of each individual effect size, the pooled Hedges’ g ranged from −0.34 to −0.20, with the lower limits of the 95% CIs ranging from −0.58 to −0.38 and the upper limits ranging from −0.09 to approximately 0.00. All pooled effects remained statistically significant, with p values ranging from 0.007 to 0.048. Heterogeneity varied across exclusions, with I^2^ ranging from 0.0% to 50.1% and τ^2^ ranging from 0.000 to 0.072. The greatest reduction in heterogeneity occurred after exclusion of Staiano et al. (2024), for which the pooled effect remained significant (Hedges’ g = −0.20, 95% CI [−0.38, −0.02], p = 0.028) and I^2^ decreased to 0.0%. Overall, the leave-one-out analyses suggested that the pooled anaerobic-capacity finding was relatively stable, although heterogeneity varied across exclusions.

### 3.5 Main effects

#### 3.5.1 Effects of mental fatigue on lower-limb explosive power

Sixteen independent effect sizes were included to assess the effect of mental fatigue on lower-limb explosive power (Martin et al., 2015; Kosack et al., 2020; Verschueren et al., 2020; Fortes et al., 2021; de Queiros et al., 2021; Weerakkody et al., 2021; Yang et al., 2022; Alix-Fages et al., 2023; Fortes et al., 2024; Gonzalez et al., 2024 (Exp 1 and Exp 2); Staiano et al., 2024; Freitas-Junior et al., 2025; Hemmat et al., 2025; Stafylidis et al., 2025; Wang et al., 2025). Heterogeneity was low (Q = 16.54, p = 0.347, I^2^ = 9.32%, τ^2^ = 0.002), suggesting that the distribution of effect sizes was relatively consistent across studies.

The pooled analysis (**Figure 5**) showed that, compared with the control condition, mental fatigue significantly reduced lower-limb explosive power (SMD = −0.16, 95% CI [−0.28, −0.03], p = 0.014). The magnitude of this effect was small, indicating a limited but statistically significant decline in performance.

**Figure 5:**
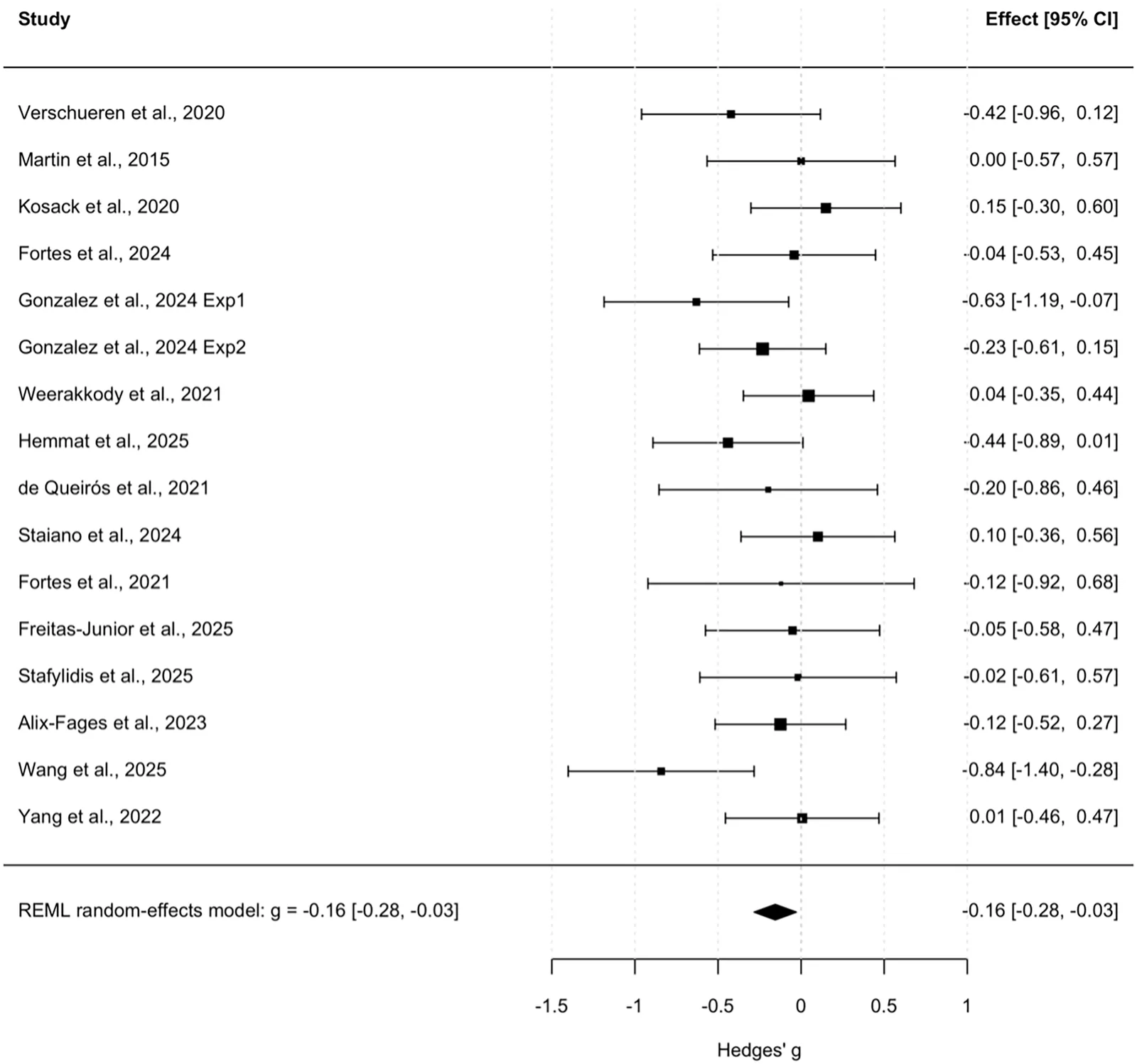
Forest plot of the effect of mental fatigue on lower-limb explosive power.

#### 3.5.2 Effects of mental fatigue on anaerobic capacity

Nine independent effect sizes were included to assess the effect of mental fatigue on anaerobic capacity (Martin et al., 2015; Kosack et al., 2020; de Queiros et al., 2021; Fortes et al., 2024; Gonzalez et al., 2024 (Exp 1 and Exp 2); Staiano et al., 2024; Evans et al., 2025; Freitas-Junior et al., 2025). Moderate heterogeneity was observed (Q = 14.02, p = 0.081, I^2^ = 43.0%, τ^2^ = 0.053), suggesting differences in participant characteristics, mental fatigue induction protocols, and anaerobic testing protocols. Therefore, a random-effects model was applied.

The pooled analysis (**Figure 6**) showed that, compared with the control condition, mental fatigue significantly reduced anaerobic capacity (SMD = −0.29, 95% CI [−0.52, −0.06], p = 0.013).

**Figure 6:**
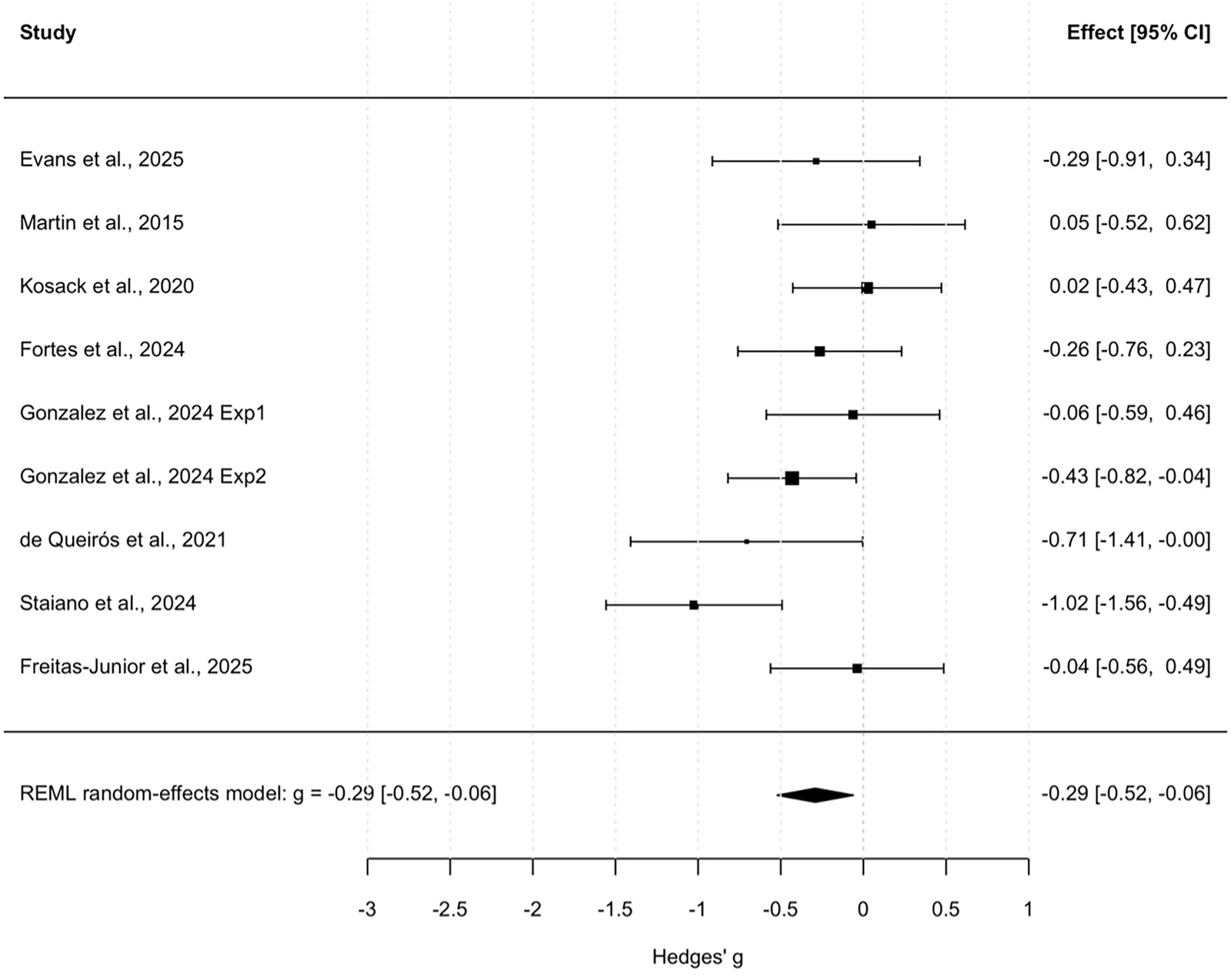
Forest plot of the effect of mental fatigue on anaerobic capacity.

### 3.6 Subgroup analysis

To explore potential sources of variation in effect sizes, subgroup analyses were conducted according to participant training level, cognitive-task type, induction duration, jump-test type, and sport specificity of anaerobic capacity tests (**Table 3**). None of the between-subgroup comparisons reached statistical significance (p > 0.05), suggesting that the current evidence is insufficient to confirm any clear moderating factor. The following results should therefore be interpreted as descriptive trends.

**Table 3:** Subgroup analyses of lower-limb explosive power and anaerobic capacity.

Outcome and subgroups	k	Pooled SMD (95% CI)	p-value	I^2^ (%)	p for subgroup difference
I. Lower-limb explosive power					
*By Athletic Level*					0.282
Non-athletes	5	−0.17 [−0.38, 0.04]	0.108	0.0	
Collegiate athletes	7	−0.27 [−0.54, −0.01]	0.040	44.9	
Youth athletes	3	−0.02 [−0.29, 0.25]	0.886	0.0	
High-level athletes	1	0.15 [−0.30, 0.60]	0.516	N/A	
*By Cognitive Task*					0.774
Stroop task	11	−0.19 [−0.36, −0.02]	0.030	36.5	
Social media	3	−0.07 [−0.37, 0.24]	0.663	0.0	
Other	2	−0.08 [−0.51, 0.34]	0.700	0.0	
*By Induction Duration*					0.975
Short (≤45 min)	11	−0.15 [−0.30, −0.01]	0.035	5.2	
Long (>45 min)	5	−0.17 [−0.45, 0.12]	0.246	33.3	
*By Jump Type*					0.751
CMJ	10	−0.12 [−0.28, 0.04]	0.153	14.1	
SJ	4	−0.18 [−0.45, 0.08]	0.174	27.6	
Other	2	−0.27 [−0.65, 0.11]	0.162	17.6	
II. Anaerobic capacity					
*By Athletic Level*					0.421
Collegiate athletes	5	−0.41 [−0.72, −0.10]	0.010	47.0	
Non-athletes	3	−0.19 [−0.58, 0.21]	0.354	32.7	
High-level athletes	1	0.02 [−0.43, 0.47]	0.922	N/A	
*By Induction Duration*					0.128
Short (≤45 min)	4	−0.51 [−0.97, −0.05]	0.030	60.0	
Long (>45 min)	5	−0.17 [−0.39, 0.04]	0.109	0.0	
*By Sport Specificity*					0.760
Sport-specific tests	6	−0.27 [−0.58, 0.05]	0.094	53.2	
Non-sport-specific tests	3	−0.35 [−0.71, 0.02]	0.062	34.8	

Subgroups with k < 3 should be interpreted descriptively.

#### 3.6.1 Subgroup analysis of lower-limb explosive power

For lower-limb explosive power, subgroup analysis by training level showed no statistically significant between-subgroup difference (p = 0.282). The collegiate-athlete subgroup showed a significant decline (k = 7, SMD = −0.27, 95% CI [−0.54, −0.01], p = 0.040, I^2^ = 44.9%), whereas the non-athlete, youth-athlete, and elite-athlete subgroups did not. Because the between-subgroup comparison was not statistically significant, the findings do not support a clear moderating effect of training level.

The between-subgroup difference by cognitive-task type was also not statistically significant (p = 0.774). The Stroop-task subgroup showed a significant negative effect (k = 11, SMD = −0.19, 95% CI [−0.36, −0.02], p = 0.030, I^2^ = 36.5%), while the social-media and other-task subgroups did not. These results do not confirm a moderating effect of cognitive-task type.

For induction duration, the short-duration subgroup (≤45 min) showed a significant negative effect (k = 11, SMD = −0.15, 95% CI [−0.30, −0.01], p = 0.035, I^2^ = 5.2%), whereas the long-duration subgroup (>45 min) did not. The between-subgroup difference was not statistically significant (p = 0.975). Therefore, the current findings do not support a dose–response relationship in which the effect of mental fatigue increases with induction duration.

None of the jump-test subgroups (CMJ, SJ, and other jump tests) showed statistically significant effects, and the between-subgroup difference was also not statistically significant (p = 0.751).

#### 3.6.2 Subgroup analysis of anaerobic capacity

For anaerobic capacity, the between-subgroup difference by athletic level was not statistically significant (p = 0.421). The collegiate-athlete subgroup showed a significant negative effect (k = 5, Hedges’ g = −0.41, 95% CI [−0.72, −0.10], p = 0.010, I^2^ = 47.0%), whereas the effects were not statistically significant among non-athletes (k = 3, Hedges’ g = −0.19, 95% CI [−0.58, 0.21], p = 0.354, I^2^ = 32.7%) or high-level athletes (k = 1, Hedges’ g = 0.02, 95% CI [−0.43, 0.47], p = 0.922). Thus, although a significant effect was observed within the collegiate-athlete subgroup, the nonsignificant between-subgroup difference does not support a clear moderating effect of athletic level.

For induction duration, the short-duration subgroup (≤45 min) showed a significant negative effect (k = 4, Hedges’ g = −0.51, 95% CI [−0.97, −0.05], p = 0.030, I^2^ = 60.0%), whereas the long-duration subgroup (>45 min) did not (k = 5, Hedges’ g = −0.17, 95% CI [−0.39, 0.04], p = 0.109, I^2^ = 0.0%). The between-subgroup difference was not statistically significant (p = 0.128). Therefore, the findings do not support a moderating effect of induction duration.

For sport specificity, neither sport-specific tests (k = 6, Hedges’ g = −0.27, 95% CI [−0.58, 0.05], p = 0.094, I^2^ = 53.2%) nor non-sport-specific tests (k = 3, Hedges’ g = −0.35, 95% CI [−0.71, 0.02], p = 0.062, I^2^ = 34.8%) showed a statistically significant pooled effect. The between-subgroup difference was also not statistically significant (p = 0.760). Thus, the current evidence does not support a clear moderating effect of sport specificity.

### 3.7 Certainty of evidence

The certainty of evidence for the two primary outcomes is summarized in **Table 4**. For lower-limb explosive power, the certainty of evidence was rated as moderate. The evidence was downgraded by one level because of methodological limitations, particularly unclear or high risk of bias related to blinding, allocation concealment, selective reporting, and crossover-specific methodological details. Inconsistency was not downgraded because statistical heterogeneity was low. For anaerobic capacity, the certainty of evidence was rated as low. The evidence was downgraded by one level for risk of bias and one level for imprecision. The pooled effect was small (Hedges’ g = −0.29, 95% CI [−0.52, −0.06]) with moderate heterogeneity (I^2^ = 43.0%). Therefore, the anaerobic-capacity finding should be interpreted with caution because of the remaining imprecision and risk of bias.

**Table 4:** Summary of findings and GRADE certainty of evidence.

Outcome	Studies	Participants	Effect estimate	Heterogeneity	Certainty of evidence	Reasons for downgrading
Lower-limb explosive power	16	292	Hedges’ g = −0.16, 95% CI [−0.28, −0.03]	I^2^ = 9.3%	moderate	Downgraded by one level for risk of bias because several studies had unclear or high risk related to blinding, allocation concealment, selective reporting, and crossover-specific methodological details.
Anaerobic capacity	9	139	Hedges’ g = −0.29, 95% CI [−0.52, −0.06]	I^2^ = 43.0%	low	Downgraded by one level for risk of bias and one level for imprecision because the 95% CI ranged from a trivial to a moderate adverse effect.

## 4 Discussion

This systematic review and meta-analysis examined the effects of mental fatigue on lower-limb explosive power and anaerobic capacity. Mental fatigue negatively affected both performance domains, although the magnitude of the effects differed. The pooled effect was small for both lower-limb explosive power and anaerobic capacity, although the effect estimate was numerically larger for anaerobic capacity. However, the corresponding certainty of evidence was moderate and low, respectively. Subgroup analyses suggested descriptive trends across participants’ training backgrounds, mental fatigue induction methods, induction durations, and exercise-testing protocols, but no clear moderators were identified.

### 4.1 General interpretation of the main effects

The main-effects analysis showed that mental fatigue significantly reduced lower-limb explosive power (Hedges’ g = −0.16, 95% CI [−0.28, −0.03], p = 0.014), with low heterogeneity (I^2^ = 9.3%) and moderate-certainty evidence, indicating a small but relatively consistent effect. Although the pooled effect remained stable across alternative correlation assumptions, the corresponding heterogeneity estimate varied, suggesting that the precise magnitude of heterogeneity should be interpreted cautiously. Anaerobic capacity was also significantly impaired (Hedges’ g = −0.29, 95% CI [−0.52, −0.06], p = 0.013), with moderate heterogeneity (I^2^ = 43.0%). Leave-one-out analysis suggested that the anaerobic-capacity finding was relatively stable, as the pooled effect remained negative and statistically significant after removal of each individual study, although the magnitude of heterogeneity varied across exclusions. Although numerically larger, the anaerobic-capacity estimate does not establish a stronger effect than that observed for lower-limb explosive power. Anaerobic-capacity tasks typically require sustained high-intensity output under greater metabolic and perceptual strain, which may plausibly increase sensitivity to mental fatigue through elevated perceived effort and impaired effort regulation (Krigolson et al., 2025).

Lower-limb explosive power typically manifests as a single maximal effort or a brief, high-force output, such as the CMJ, SJ, or vertical jump. These tasks are extremely brief and rely primarily on rapid motor-unit recruitment, neuromuscular coordination, and energy from the phosphagen system. Their movement patterns are relatively fixed, and participants may complete them with a high degree of motor automaticity (Gastin, 2001). Therefore, although the effect of mental fatigue on these tasks was statistically significant, its magnitude was small. Mental fatigue may cause a limited but consistent impairment in explosive power by reducing attention, weakening neural readiness before maximal effort, or slightly disrupting motor-cortex excitability (Boksem et al., 2005).

In contrast, anaerobic capacity tasks typically include the Wingate test, repeated sprints, sport-specific shuttle runs, or continuous jumps. These tasks require both high power output and sustained high-intensity effort despite increased metabolic stress, muscular discomfort, and respiratory load (Driss and Vandewalle, 2013; Astokorki and Mauger, 2017). Anaerobic capacity may therefore depend more strongly on sustained motivation, subjective-effort regulation, and tolerance of discomfort. Mental fatigue may prematurely increase perceived exertion, causing a decline in speed, power output, or movement quality before peripheral physiological limits are reached. These mechanisms may provide a plausible explanation for the adverse effects of mental fatigue on sustained high-intensity performance (Pageaux, 2014).

### 4.2 Participant characteristics

Negative effects were observed within certain training-level subgroups, but the between-subgroup differences were not statistically significant. It is therefore not possible to conclude that any particular group is more susceptible to mental fatigue. Long-term training may enhance motor automaticity and neuromuscular-control efficiency, reducing athletes’ reliance on prefrontal cognitive resources during skilled movements such as jumps and sprints (Poldrack et al., 2005; Zhang et al., 2019). At the same time, more highly trained participants may mobilize greater subjective effort during high-intensity tasks and push closer to their physiological limits. In repeated sprints, consecutive jumps, or other fatiguing anaerobic tasks, increased perceived effort, reduced motivation, or impaired sustained-effort regulation may therefore be more apparent. The relationship between training level and the effects of mental fatigue may not be linear and may depend on the exercise task and testing context (Marcora et al., 2009; Pageaux, 2014).

### 4.3 Mental fatigue induction protocol

The Stroop-task and short-duration induction subgroups showed significant negative effects for some outcomes; however, between-subgroup differences in cognitive-task type and induction duration were not statistically significant. The current findings therefore do not demonstrate that the Stroop task is more potent than other cognitive tasks or support a dose–response relationship in which the effects of mental fatigue increase with induction duration. The impact of mental fatigue may depend not only on task duration but also on cognitive-load characteristics, task novelty, individual engagement, and subjective fatigue after induction (O’Keeffe et al., 2020; Dallaway et al., 2022; Behrens et al., 2023; Pickering et al., 2024). For example, the Stroop task involves response inhibition and cognitive conflict, which may induce fatigue related to attentional control and executive function. Social media use may be more ecologically valid, but its cognitive-load intensity, emotional stimuli, and individual usage patterns vary considerably, potentially leading to inconsistent induction effects (Fortes et al., 2021, 2024; Freitas-Junior et al., 2025). Future studies should combine subjective fatigue, cognitive-task performance, and neurophysiological indicators rather than relying solely on induction duration.

### 4.4 Exercise testing protocol

Regarding lower-limb explosive power, the CMJ, SJ, and other jump-test subgroups did not show statistically significant effects, and the between-subgroup differences were also not statistically significant. Thus, the current evidence does not indicate that jump-test type moderates the effect of mental fatigue on lower-limb explosive power. These tests are brief, involve relatively fixed movement patterns, and rely primarily on rapid neuromuscular recruitment (Gastin, 2001; Cormie et al., 2011; Petrigna et al., 2019).

Regarding anaerobic capacity, neither the sport-specific nor the non-sport-specific subgroup showed a statistically significant pooled effect, and the between-subgroup difference was also not statistically significant. Although sport-specific tests may impose greater attentional and motor-coordination demands, the current evidence does not support sport specificity as a moderator of the effect of mental fatigue on anaerobic capacity (Trecroci et al., 2020; Habay et al., 2021; Yuan et al., 2023).

### 4.5 Strengths and limitations

This study focuses on the effects of mental fatigue on lower-limb explosive power and anaerobic capacity. By isolating these two types of high-intensity performance from general athletic performance and quantitatively combining them, the study facilitates a more precise understanding of the role of mental fatigue in short-duration, high-intensity exercise. Additionally, this study included relevant research published in recent years and conducted subgroup analyses based on variables such as training level, cognitive task type, induction time, jump test type, and sport specificity of the test, providing a reference for interpreting differences in research findings. The certainty of evidence was also assessed using the GRADE approach. Evidence was rated as moderate for lower-limb explosive power and low for anaerobic capacity. The lower certainty for anaerobic capacity was mainly due to risk of bias and imprecision.

This study has several limitations. The number of included studies was relatively limited, and some subgroups contained few studies, affecting the stability of the subgroup analyses. Although significant within-subgroup effects were observed for some subgroup categories, none of the between-subgroup comparisons was statistically significant. These findings should therefore be interpreted as descriptive rather than confirmatory evidence of moderation. Differences in participant characteristics, mental-fatigue induction protocols, and exercise-testing methods may have contributed to the remaining heterogeneity in anaerobic-capacity outcomes and affected the stability of the pooled results. Furthermore, strict blinding is difficult to achieve in mental fatigue interventions, and some studies insufficiently reported methodological details related to randomization, allocation concealment, and prespecified outcome reporting. The current evidence primarily comes from young and predominantly male samples; studies involving female athletes, elite athletes, and different age groups remain scarce. Caution is therefore warranted when generalizing these findings. In addition, potential mechanisms involving perceived effort, motivation, and effort regulation were not quantitatively synthesized in the present meta-analysis; therefore, these mechanisms should be interpreted as theoretical explanations rather than mechanisms directly established by the pooled evidence.

### 4.6 Future research directions

The mechanisms underlying the effects of mental fatigue on high-intensity performance remain unclear. Future studies should further investigate how mental fatigue affects different neuromuscular systems. Neuroimaging and electrophysiological techniques, such as electroencephalography (EEG), functional near-infrared spectroscopy (fNIRS), and transcranial magnetic stimulation (TMS), may help identify neurobiological markers associated with cognitive-load-induced changes in cortical excitability.

Future research should also examine how ecologically valid forms of mental fatigue, such as pre-competition tactical-analysis meetings, high-pressure esports activities, and prolonged use of specific social-media applications, affect athletic performance by disrupting specific cognitive domains. Studies focusing on female athletes and individual differences in cognitive tolerance are also important directions for future research.

## 5 Conclusion

This systematic review and meta-analysis suggests that mental fatigue negatively affects both lower-limb explosive power and anaerobic capacity. The effect on lower-limb explosive power was small but statistically significant, while anaerobic capacity was also significantly impaired. However, the anaerobic-capacity finding should be interpreted cautiously because of imprecision and low-certainty evidence. Given the differing certainty of evidence across outcomes and the predominance of young male participants in the included studies, the practical implications should be interpreted cautiously. Coaches and athletes may consider minimizing unnecessary cognitive load before training or competition involving explosive or high-intensity performance, although the generalizability of this recommendation across sexes, age groups, and training levels remains uncertain. Future studies should use standardized mental-fatigue protocols, clearly defined performance outcomes, adequately powered randomized crossover designs, and more diverse samples across sex, age, training levels, and sport disciplines.

## Data Availability

The original contributions presented in the study are included in the article/**Supplementary Material**. Further inquiries can be directed to the corresponding author.
